# Clinical significance of NGAL and KIM-1 for acute kidney injury in patients with scrub typhus

**DOI:** 10.1371/journal.pone.0175890

**Published:** 2017-04-18

**Authors:** In O. Sun, Sung Hye Shin, A. Young Cho, Hyun Ju Yoon, Mi Yok Chang, Kwang Young Lee

**Affiliations:** 1 Division of Nephrology, Department of Internal Medicine, Presbyterian Medical Center, Jeonju, Korea; 2 Christian Medical Research Center, Presbyterian Medical Center, Jeonju, Korea; 3 Division of Infection, Department of Internal Medicine, Presbyterian Medical Center, Jeonju, Korea; University of Sao Paulo Medical School, BRAZIL

## Abstract

**Background:**

The aim of this study is to investigate the clinical significance of neutrophil gelatinase–associated lipocalin (NGAL) and kidney injury molecule-1 (KIM-1) for acute kidney injury (AKI) in patients with scrub typhus.

**Methods:**

From 2014 to 2015, 145 patients were diagnosed with scrub typhus. Of these, we enrolled 138 patients who were followed up until renal recovery or for at least 3 months. We measured serum and urine NGAL and KIM-1 levels and evaluated prognostic factors affecting scrub typhus–associated AKI.

**Results:**

Of the 138 patients, 25 had scrub typhus–associated AKI. The incidence of AKI was 18.1%; of which 11.6%, 4.3%, and 2.2% were classified as risk, injury, and failure, respectively, according to RIFLE criteria. Compared with patients in the non-AKI group, patients in the AKI group were older and showed higher total leukocyte counts and hypoalbuminemia or one or more comorbidities such as hypertension (72% vs 33%, p<0.01), diabetes (40% vs 14%, p<0.01), or chronic kidney disease (32% vs 1%, p<0.01). In addition, serum NGAL values (404± 269 vs 116± 78 ng/mL, P<0.001), KIM-1 values (0.80± 0.52 vs 0.33± 0.68 ng/mL, P<0.001), urine NGAL/creatinine values (371± 672 vs 27± 39 ng/mg, P<0.001) and urine KIM-1/creatinine values (4.04± 2.43 vs 2.38± 1.89 ng/mg, P<0.001) were higher in the AKI group than in the non-AKI group. By multivariate logistic regression, serum NGAL and the presence of chronic kidney disease were significant predictors of AKI.

**Conclusion:**

Serum NGAL might be an additive predictor for scrub typhus–associated AKI.

## Introduction

Scrub typhus, which is caused by *Orientia tsutsugamushi* and is transmitted by chigger bite, is an acute febrile disease that can involve many vital organs including the kidneys [1–3]. The incidence of acute kidney injury (AKI) in scrub typhus has been reported to range from 21 to 43% [4–6], and old age and co-morbidity are associated with the development of AKI [6]. The possible mechanisms of AKI associated with scrub typhus include pre-renal failure, septic shock, vasculitis, rhabdomyolysis, and direct renal invasion of *O*. *tsutsugamushi* [7–11].

Acute kidney injury is a common disorder that is strongly linked to short- and long-term morbidity and mortality [12, 13]. More timely diagnosis would allow for earlier intervention and improve patient outcomes. Many investigations have sought to identify markers that can facilitate the early diagnosis, differential diagnosis, and short- and long-term prognosis of AKI. Several novel AKI biomarkers have been identified including neutrophil gelatinase–associated lipocalin (NGAL), kidney injury molecule 1 (KIM-1), cystatin C, interleukin (IL)-18, and liver-type fatty acid–binding protein [14–17]. Of these, NGAL and KIM-1 are the most widely studied biomarkers of AKI. However, there is no report on the relationship between scrub typhus-associated AKI and biomarkers such as NGAL and KIM-1.

Therefore, we investigated the clinical significances of neutrophil gelatinase–associated lipocalin (NGAL) and kidney injury molecule-1 (KIM-1) for AKI in patients with scrub typhus.

## Methods

### Patient selection

Between 2014 and 2015, 145 patients were diagnosed with scrub typhus, which was confirmed by a positive IgM ELISA for scrub typhus (InBios International Inc., Seattle, WA, USA) in patients with acute febrile illness and rash. Patients who were transferred to another hospital during treatment or had concomitant infections such as leptospirosis, malaria, or dengue fever were excluded from the study. We also excluded patients who were not followed up until complete recovery of renal function or for at least 3 months after discharge. After exclusions, a total of 138 patients were enrolled in this study after obtaining written informed consent from each participant. This study was approved by the Institutional Review Board of the Presbyterian Medical Center, Jeonju, South Korea.

All patients underwent a detailed clinical history and examination, a standard set of investigations including complete blood counts, liver function tests, serum creatinine, urea, electrolytes, chest radiograph, three peripheral blood smears for malaria, urinalysis, and two blood cultures. The definition of AKI was based on the RIFLE (Risk, Injury, Failure, Loss of kidney function, and End-stage kidney disease) criteria [18], and patients were categorized into the R, I or F categories. The estimated glomerular filtration rate (eGFR) was calculated using the abbreviated Modification of Diet in Renal Disease (MDRD) equation [19]. When the baseline serum creatinine was not available, it was calculated using the standard four-variable MDRD formula assuming eGFR of 75 mL/min/1.73 m^2^. The RIFLE class was determined based on the worst among serum creatinine levels, eGFR, and urine output criteria. Renal replacement therapy was initiated using the standard indications.

### Sandwich enzyme-linked immunosorbent assay analysis of NGAL and KIM-1 expression in serum and urine samples

Blood and urine samples were collected at the first presentation, before specific treatment, and at follow-up (3 days after taking the initial sample). Peripheral venous blood was drawn into pyrogen-free vacuum blood collection tubes with EDTA as anticoagulant, centrifuged within 30 minutes at 2,000 g for 5 minutes to obtain platelet-poor plasma, and the obtained samples were stored in multiple aliquots at 280uC until analysis. The samples were then stored at –80°C until assayed. Random spot urine samples were also centrifuged at 2,000 g for 5 min and the supernatants were stored in aliquots at –80°C. Plasma and urine NGAL and KIM-1 levels were measured using the human NGAL enzyme-linked immunosorbent assay (ELISA) kit (R&D Systems, Minneapolis, MN, USA) and the KIM-1 ELISA kit (R&D Systems, Minneapolis, MN, USA). The urinary creatinine (Cr) concentration was used to normalize NGAL and KIM-1 measurements to account for the influence of urinary dilution on concentration. Urinary levels of the biomarkers were expressed as uNGAL/Cr and uKIM-1/Cr ratio as ng/mg creatinine. Interassay and intraassay coefficient variations were 5% to 10% for batched samples analyzed on the same day.

### Multiplex assays

Interferon (IFN) γ, tumor necrosis factor [TNF]-α, and TH2 (IL-10) cytokines were measured with multiplex bead flow cytometry (Flowcytomix; Bender Medsystems, Vienna, Austria). Briefly, patient serum was added to the bead mixtures that were coated with antibodies to human cytokines. A biotin-conjugated mixture was added to the serum antibody-bead mixture complex. After incubation, a streptavidin-phycoerythrin (PE) solution was added to bind the biotin-conjugate. Following further incubation, unbound streptavidin-PE was removed during a subsequent wash step and the analyte was coupled with bead mixtures, biotinylated antibody, and streptavidin-PE emitting fluorescent signals. A standard curve was prepared from serial dilutions of a standard mixture. The concentrations of human cytokines were analyzed by FACScanto flow cytometry (Becton Dickinson Biosciences, San Jose, CA, USA) and Flowcytomix Pro 2.2 software (Bender Medsystems).

### Statistical analysis

All data are presented as mean ± standard deviation unless otherwise specified. The baseline characteristics of patients in the non-AKI and AKI groups were compared using *t* tests, chi-square test, or Fisher’s exact test, as appropriate. Clinically relevant parameters or variables that were significantly associated with the presence of AKI in the univariate analysis were included in the multivariate analysis. Receiver operating characteristics (ROC) analysis was used to calculate the area under the curve (AUC) for NGAL and KIM-1 and to find the best cutoff values for the diagnosis of AKI. A P-value <0.05 was considered statistically significant. Statistical analysis was carried out using SPSS version 22.0.

## Results

### Baseline characteristics

The baseline characteristics of the 138 study subjects are presented in Table 1.

**Table 1:** The clinical and laboratory findings of the 138 patients with scrub typhus.

Characteristics	
Age, years	65 ± 13
Male, n (%)	49 (36)
Eschar, n (%)	130 (94)
Co-morbidity, n (%)	64 (46)
Diabetes, n (%)	26 (19)
Hypertension, n (%)	55 (40)
CKD, n (%)	9 (7)
Duration of hospital stay, days	6.4 ± 4.3
Fever, n (%)	132 (96)
Systolic BP (<90 mmHg), n (%)	8 (6)
ICU care, n (%)	5 (4)
Serum creatinine (mg/dl)	1.07 ± 0.58
eGFR _adm_, ml/min/1.73m^2^	64 ± 28
Serum ALT (IU/L)	82 ± 124
Total leukocyte count (× 10^3^/ mL)	7.73 ± 5.96
Platelet count (× 10^3^/ mL)	134 ± 55
Patient with baseline renal function, n (%)	73 (53)
Acute kidney injury, n (%) RIFLE_SCr_ RIFLE_UO_ RIFLE_SCr+UO_	25 (18) 24 (17) 7 (5) 25 (18)

The patients included 49 (36%) men and 89 (64%) women with a mean age of 65 years (range, 20–86). Eschars were found in 130 (94%) patients. Sixty-four patients had comorbidities such as hypertension, diabetes, or chronic kidney disease. The mean duration of hospital stay was 6.4 days, and 132 (96%) patients had fever during the hospitalization period. Eight (6%) patients were hypotensive (systolic blood pressure <80 mmHg) at admission, and five (4%) required admission to the intensive care unit. The mean plasma alanine aminotransferase (ALT) concentration was 82 IU/L, and the mean total leukocyte count was 7.73×10^3^/mL. The mean eGFR was 64mL/min/1.73m^2^. Of the 138 patients enrolled in this study, 25 (18%) experienced AKI. All patients were treated with anti-rickettsia agents such as doxycycline or azythromycin. Upon admission, blood and urine samples were collected from all patients before treatment. Second blood and urine samples (3 days after the initial sample) were analyzed in 37 patients.

### Comparison of clinical characteristics between non-AKI group and AKI group

Compared with patients in the non-AKI group, patients in the AKI group were older (74 ± 9 vs. 63 ± 12 years, P<0.01) and more likely to have one or more comorbidity such as diabetes (40% vs. 14%, P<0.01), hypertension (72% vs. 33%, P<0.01), or chronic kidney disease (32% vs. 1%, P<0.01) (Table 2).

**Table 2:** Comparison of baseline characteristics between non-AKI and AKI group.

	AKI (n = 25)	Non-AKI (n = 113)	P-value
Age	74 ± 9	63± 12	< 0.01
Male, n(%)	12 (48)	37 (33)	NS
Duration of hospital stay, days	5.6 ± 2.5	10.2 ± 7.7	< 0.01
Comorbidity, n(%) Diabetes, n (%) Hypertension, n (%) CKD, n (%)	18 (72) 10 (40) 18 (72) 8 (32)	46 (41) 16 (14) 37 (33) 1 (1)	< 0.01 < 0.01 < 0.01 < 0.01
Systolic BP (<90 mmHg), n (%)	5 (20)	3 (3)	< 0.01
Hemoglobin (mg/dl)	11.20 ± 1.80	13.12 ± 1.60	< 0.01
Leukocyte (× 10^3^/ mL)	11.00 ± 4.78	7.00 ± 5.97	< 0.01
Platelet count (× 10^3^/ mL)	123 ± 67	137 ± 52	NS
Total bilirubin level	0.81 ± 0.45	0.76 ± 0.36	NS
Serum albumin (mg/dl)	3.27 ± 0.50	3.78 ± 0.56	< 0.01
Serum ALT (IU/L)	56 ± 31	88 ± 136	NS
C-reactive protein (mg/dl)	9.28 ± 5.71	5.73 ± 4.04	< 0.01
Creatinine (mg/dl)	1.94 ± 0.91	0.87 ± 0.17	< 0.01
eGFR ml/min/1.73m^2^	28 ± 13	72 ± 24	< 0.01
Serum NGAL (ng/mL)	404 ± 269	116 ± 78	< 0.01
Urine NGAL/Cr (ng/mg)	371 ± 672	27± 39	< 0.01
Serum KIM-1 (ng/mL)	0.80 ± 0.52	0.33 ± 0.68	< 0.01
Urine KIM-1/Cr (ng/mg)	4.04 ± 2.43	2.38 ± 1.89	< 0.01
IL-10 (pg/mL)	152 ± 163	62 ± 103	< 0.01
TNF-α (pg/mL)	55 ± 58	15 ± 15	< 0.01
IFN (pg/mL)	169 ± 297	156 ± 278	NS

Patients in the AKI group had worse renal function (28 ± 13 vs. 72 ± 24 mL/min/1.73m^2^, P<0.01) at admission, and a higher incidence of hypotension while in the hospital. Inflammatory markers such as total leukocyte count were higher in patients with AKI than in those without AKI (11.00 × 10^3^/ mL vs. 7.00 × 10^3^/mL, P<0.01). Plasma ALT concentrations and total bilirubin levels were not different between the two groups. Serum NGAL (404 ± 269 vs. 116 ± 78 ng/mL, P<0.01), KIM-1 (0.80 ± 0.52 vs. 0.33± 0.68 ng/mL, P<0.01), urine NGAL/creatinine values (371± 672 vs. 27± 39 ng/mg, P<0.01), and urine KIM-1/creatinine values (4.04 ± 2.43 vs. 2.38 ± 1.89 ng/mg, P<0.01) were higher in the AKI group than in the non-AKI group. Plasma levels of IL-10 (152 ± 163 vs. 62 ± 103 pg/mL, P<0.01) and TNF-α (55 ± 58 vs. 15 ± 15 pg/mL, P<0.01) were higher in the AKI group than in the non-AKI group, but there was no difference in IFN-γ levels between the two groups. Although there was no difference in NGAL and KIM-1 levels between the first and second samples in the non-AKI group (n = 26), the values decreased during treatment in the AKI group (n = 11).

### Clinical course of acute kidney injury in patients with scrub typhus

According to the RIFLE criteria, 16 (64%), 6 (24%), and 3 (12%) patients in our study fell into the R, I, and F categories, respectively (Table 3).

**Table 3:** Clinical characteristics of 25 patients with AKI.

		Risk (n = 16)	Injury (n = 6)	Failure (n = 3)
RIFLE criteria	RIFLE_SCr_ (n = 24)	16 (67)	5 (21)	3 (12)
	RIFLE_UO_ (n = 7)	2 (29)	2 (29)	3 (42)
	RIFLE_SCr+UO_ (n = 25)	16 (64)	6 (24)	3 (12)
NGAL	Serum NGAL (ng/mL)	328± 270	530 ± 266	559 ± 125
	Urine NGAL/Cr (ng/mg)	170 ± 392	280 ± 190	1629 ± 1206
KIM-1	Serum KIM-1 (ng/mL)	0.69 ± 0.57	0.88 ± 0.35	1.23 ± 0.37
	Urine KIM-1/Cr (ng/mg)	3.36 ± 0.57	4.05 ± 0.35	7.68 ± 0.37
Cytokines	IL-10 (pg/mL)	86 ± 91	228 ± 174	338 ± 224
	TNF-α (pg/mL)	50 ± 58	44 ± 42	104 ± 82
	IFN (pg/mL)	154 ± 303	214 ± 362	162 ± 192
**FENa < 1%, n (%)**		15 (94)	3 (50)	1 (33)
**Recovery of renal function within 72 h, n (%)**		16 (100)	3 (50)	0

**Fig 1:**
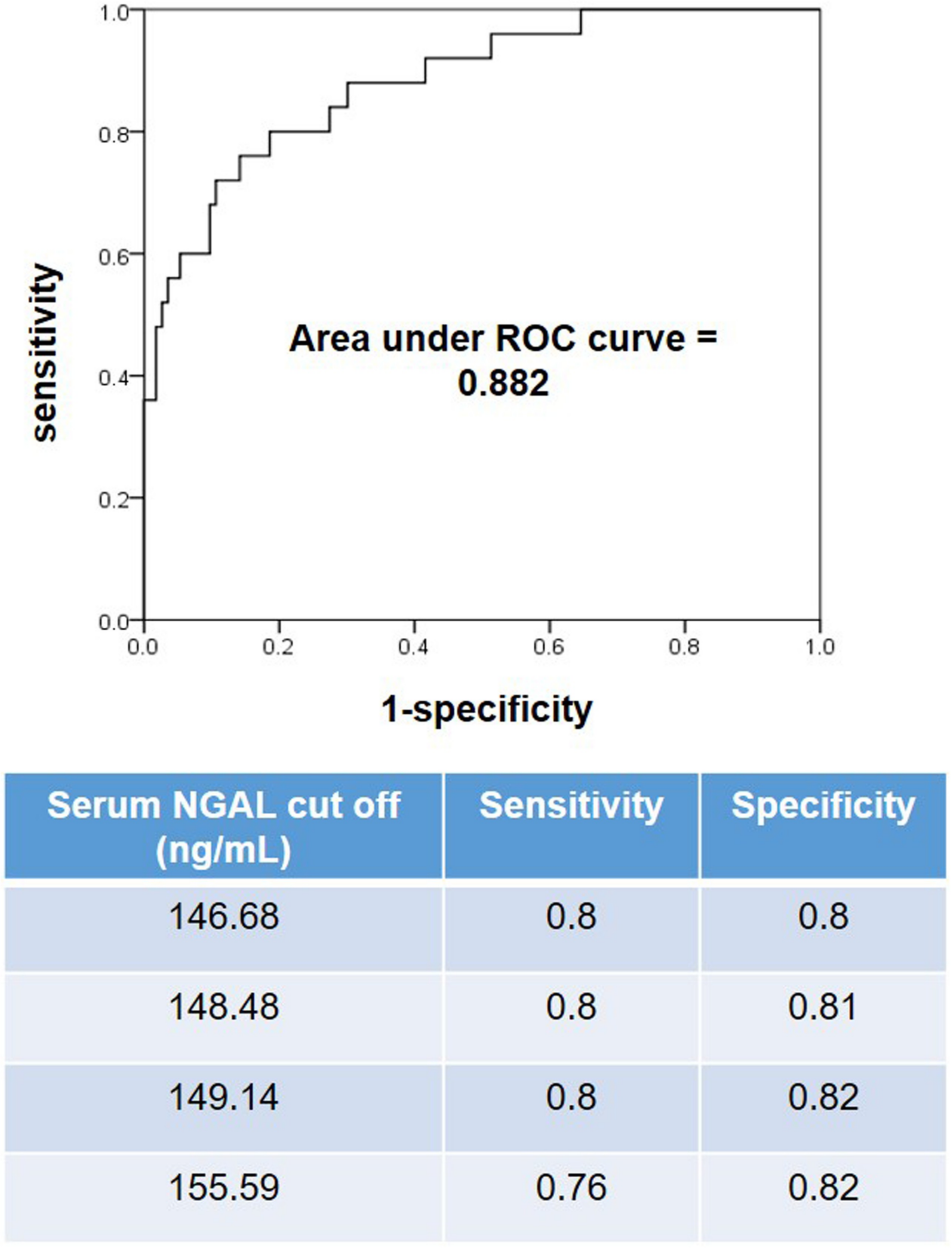
Receiver operating characteristic curve and performance characteristics for serum NGAL upon admission. The area under the ROC curve for the serum NGAL test is 88% (CI 0.808–0.956).

The NGAL and KIM-1 levels in serum and urine increased progressively with AKI severity based on the RIFLE criteria. Twenty-two patients had AKI prior to admission and three patients experienced AKI during their hospitalization. Nineteen patients recovered from AKI within 72 hours. Twenty-four patients with AKI recovered within 3 months without renal replacement therapy and one patient died due to septic shock from pneumonia. Using univariate analysis, age, diabetes, hypertension, chronic kidney disease, occurrence of at least one episode of hypotension, hemoglobin, total leukocyte count, serum albumin, serum NGAL, urine NGAL/creatinine, serum KIM-1, urine KIM-1/creatinine, IL-10, and TNF-α were significant predictors of AKI. After adjustment for these factors in a multivariate logistic regression analysis, the presence of chronic kidney disease and serum NGAL were identified as the only significant predictors of AKI (Table 4). The area under the ROC curve was 0.882 for serum NGAL. Serum NGAL of 149.14 ng/mL yielded good sensitivity (80%) and specificity (82%) (Fig 1).

**Table 4:** Predictors of AKI (multivariative analysis).

Variables	Relative risk	95% confidence interval Lower Upper	P value
**Serum NGAL**	**1.04**	**1.003 1.019**	**0.006**
**Presence of CKD**	**67.01**	**2.120 2118.817**	**0.017**

## Discussion

In the present study, we show that serum and urine levels of NGAL and KIM-1 are higher in patients with AKI than in those without AKI, and that serum NGAL and the presence of chronic kidney disease are significant predictors of AKI in scrub typhus. Therefore, serum NGAL could be an additive predictor for scrub typhus-associated AKI. Our findings provide a rationale for using NGAL as a diagnostic tool for the detection of AKI in patients with scrub typhus.

Scrub typhus is endemic to geographically distinct regions, which include Japan, Taiwan, China, and South Korea. It can cause multiorgan complications including AKI, and the incidence of AKI ranges from 21 to 43% [4–6]. In this study, the incidence of AKI was 18%, which was similar to previous results. However, the incidences were different according to the serum creatinine (17%) or urine output (5%) criteria. Because the patients with initial normal serum creatinine were not placed urinary catheter in presenting study, we believe that the incidence of AKI based on RIFLE_UO_ might be underestimated. After more than a decade of intensive research efforts, several novel AKI biomarkers have been identified and their performance has been validated for the prediction of AKI in in various settings such as pediatric and adult cardiac surgery patients, critically ill patients, and patients in the emergency room, as well as in the kidney transplant setting [20–23]. However, to date, there have been no reports of evaluation of NGAL and KIM-1 in patients with scrub typhus.

We used RIFLE classification system in the presenting study instead of the Acute Kidney Injury Network (AKIN) classification or the Kidney Disease: Improving Global Outcome (KDIGO) AKI classification system. The major strength of RIFLE classification system is that it considers the change of any measure of renal function from baseline [24]. Given the absence of readily available methods for the measurement of GFR when serum creatinine concentration is not in a steady state, changes in GFR are not included in the AKIN or KDIGO AKI classification systems [24–26]. Baseline renal function was not available in 65 (47%) patients in this study. Furthermore, the diagnostic criteria for AKI by using AKIN and KDIGO system require at least 2 creatinine values within 48 hours. However, most patients with AKI in scrub typhus have the worst renal function at admission [6], which was also shown in this study. Therefore, we used the RIFLE classification system in this study instead of the AKIN or KDIGO criteria.

In the present study, we measured serum and urine NGAL and KIM-1 of patients with scrub typhus upon admission before treatment and found that NGAL and KIM-1 levels in both serum and urine were higher in the AKI group than in the non-AKI group. The NGAL and KIM-1 levels in serum and urine seemed to be correlated with AKI severity based on RIFLE criteria (R<I<F), and reduction of NGAL and KIM-1 levels was shown during serial measurement following treatment. Furthermore, serum NGAL and the presence of chronic kidney disease were significant predictors of AKI in scrub typhus. Therefore, we believe that serum NGAL could be an additive predictor for scrub typhus–associated AKI, as shown for other types of AKI in previous studies.

However, we question whether serum NGAL is clinically useful for the detection of AKI in patients with scrub typhus due to the characteristics of AKI in this setting. In general, biomarkers such as NGAL are valuable when they can detect AKI before the increase in plasma creatinine, thus allowing for earlier intervention. However, in our study 22 out of 25 patients with AKI had worsening of renal function prior to admission, similar to previous results [6]. Thus, AKI biomarkers, including serum NGAL, might be less useful in scrub typhus than in other clinical settings, because most patients with AKI associated with scrub typhus already had AKI prior to admission. However, three patients in our study experienced AKI within 72 hours after admission, and for these patients the mean serum NGAL levels (276 vs. 116 ng/mL) on admission were higher than those were for patients in the non-AKI group. Therefore, serum NGAL might be helpful to detect AKI in patients with scrub typhus in cases that develop during hospitalization. To clarify the utility of serum NGAL, studies with a larger number of scrub typhus participants that develop AKI during hospitalization are needed.

It was previously reported that NGAL could predict death or dialysis at the time of admission [26–28]. However, in this study, only one patient died, and no patients needed renal replacement therapy. The patient who died had AKI during hospitalization, which improved within several days. The cause of death was septic shock resulting from the aggravation of hospital-acquired pneumonia on the 45^th^ hospital day. Therefore, we cannot estimate whether NGAL is a predictor of death or dialysis at the time of admission in scrub typhus–associated AKI.

The pathogenesis of *O*. *tsutsugamushi* infection involves initial infection of endothelial cells and subsequent perivascular infiltration of T cells and monocyte/macrophages, resulting in vasculitis [29, 30]. This interaction between microbes and endothelial cells triggers a wide range of inflammatory responses, including the production of several cytokines by endothelial and non-endothelial cells. It was reported that some cytokines, including IL-8, contribute to disease severity in patients with scrub typhus [31], and cytokines such as IL-6 and IL-10 were associated with the development of AKI [32]. In our study, levels of three cytokines, IL-10, TNF, and INF, were higher in the AKI group than the non-AKI group. However, these cytokines were not identified to be predictors of AKI in patients with scrub typhus. Washburn et al. reported that urinary IL-18 concentration increases in 24–48 hours prior to AKI based on the RIFLE criteria and is correlated with AKI severity [33]. In the present study, even though the results are statistically significant, we noted a trend of the correlation of IL-10 concentration (R<I<F). To reveal the relationship between AKI severity and cytokine more clearly, studies with a larger number of blood and urine samples to measure various cytokines are needed.

Our study has some limitations. First, the number of patients in the AKI group was too small and more studies with a larger population are needed. Second, we measured AKI biomarkers serially in only 37 patients. More samples are needed to evaluate the change in NGAL and KIM-1 more precisely.

To our knowledge, this is the first report to show the relationship between AKI biomarkers and scrub typhus. In our study, the incidence of scrub typhus–associated AKI was 18%, and serum NGAL and the presence of chronic kidney disease were significant predictors of AKI in scrub typhus. Therefore, we think that serum NGAL might be a helpful biomarker for the detection of AKI in patients with scrub typhus.

## Supporting information

S1 File The minimal data set, including the study population characteristics, in this study (XLSX)

## Abbreviations

NGAL: neutrophil gelatinase-associated lipocalin
KIM-1: kidney injury molecule-1
AKI: acute kidney injury
IL: interleukin
IFN: interferon
TNF: tumor necrosis factor
eGFR: estimated glomerular filtration rate
eGFR _adm_: eGFR at the time of admission
MDRD: Modification of Diet in Renal Disease
ALT: alanine aminotransferase
CKD: chronic kidney disease
BP: blood pressure
ICU: intensive care unit
FENa: fractional excretion of sodium
RIFLE_Scr_: RIFLE diagnosis based on RIFLE serum creatinine criteria only
RIFLE_UO_: RIFLE diagnosis based on RIFLE urine output criteria only
RIFLE_Scr+UO_: RIFLE diagnosis based on both serum creatinine and urine criteria
